# Genetic variants in patients with multiple arterial aneurysms

**DOI:** 10.1007/s00423-024-03488-5

**Published:** 2024-10-09

**Authors:** Daniel Körfer, Caspar Grond-Ginsbach, Andreas S. Peters, Sebastian Burkart, Maja Hempel, Christian P. Schaaf, Dittmar Böckler, Philipp Erhart

**Affiliations:** 1 Department of Vascular and Endovascular Surgery, Heidelberg University Hospital, Im Neuenheimer Feld 420, 69120 Heidelberg, Germany; 2 Vascular Biomaterialbank Heidelberg (VBBH), Heidelberg University Hospital, Heidelberg, Germany; 3 Institute of Human Genetics, Heidelberg University, Heidelberg, Germany

**Keywords:** Aortic aneurysm, Peripheral arterial aneurysm, Genetic variants, Genetic testing, Exome sequencing, Loeys-Dietz syndrome, Ehlers-Danlos syndrome

## Abstract

**Purpose:**

The aim of this study was to identify causal genetic variants in patients with multiple arterial aneurysms.

**Methods:**

From a total cohort of 3107 patients diagnosed with an arterial aneurysm from 2006 to 2016, patients with known hereditary connective tissue diseases, vasculitis, or other arterial pathologies (*n* = 918) were excluded. Of the remaining cohort (*n* = 2189), patients with at least 4 aneurysms at different arterial locations (*n* = 143) were included. Nine blood samples of respective patients were available and derived from the institutional vascular biomaterial bank, and analyzed by whole exome sequencing (WES). Possible candidate variants were selected based on in silico predictions: (I) Truncating variants or (II) Variants that were classified as likely pathogenic (SIFT score < 0.05 or PolyPhen score > 0.9) and with low (< 0.001) or unknown gnomAD allele frequency. The human genome databases GeneCards and MalaCards were used to correlate the variants with regard to possible associations with vascular diseases.

**Results:**

A total of 24 variants in 23 different genes associated with vascular diseases were detected in the cohort. One patient with eight aneurysms was heterozygous for a variant in *SMAD3*, for which pathogenic variants are phenotypically associated with Loeys-Dietz syndrome 3. A heterozygous variant in *TNXB* was found in a patient with five aneurysms. Homozygous or compound heterozygous pathogenic variants in this gene are associated with Ehlers-Danlos syndrome (classical-like). Another patient with six aneurysms carried two heterozygous *TET2* variants together with a heterozygous *PPM1D* variant. Pathogenic variants in these genes are associated with clonal hematopoiesis of indeterminate potential (CHIP), a known risk factor for cardiovascular disease.

**Conclusion:**

All nine patients in this study carried variants in genes associated with vascular diseases. Current knowledge of the specific variants is insufficient to classify them as pathogenic at the present time, underlining the need for a better understanding of the consequences of genetic variants. WES should be considered for patients with multiple arterial aneurysms to detect germline variants and to improve clinical management for the individual and family members.

**Supplementary Information:**

The online version contains supplementary material available at 10.1007/s00423-024-03488-5.

## Introduction

Arterial aneurysms are defined by an enlargement of 150% of the original vessel diameter. Arterial wall dilation is caused by chronic inflammatory alteration and can lead to vessel rupture or thromboembolic events with fatal consequences for affected patients. The abdominal aortic aneurysm (AAA) is the most frequent aneurysmal localization, with an estimated prevalence between 2-8% in developed countries and reported mortalities of 15,000 deaths per year in the United States, 6000 to 8000 deaths per year in the United Kingdom and 1250 deaths per year in Germany [1, 2]. Aneurysm formation is strongly associated with atherosclerosis. Chronic inflammatory processes alter the wall constitution accompanied by degradation of extracellular matrix and apoptosis of smooth muscle cells, leading to decreased vessel wall elasticity and stability [3]. As genetic factors are discussed for abdominal aortic aneurysm formation, genetic analysis especially in patients with thoracic aortic aneurysm and dissection (TAAD) can reveal individual risk and influence therapeutic management [4].

Patients with multiple arterial aneurysms show clinical and histopathological differences to patients with a single AAA, with an earlier disease onset, distinct peripheral aneurysm locations and potentially different inflammatory pathogenesis [5, 6]. Moreover, clinical outcomes seem to differ from patients with single aneurysms [7, 8]. Based on these observations, we suspect the influence of underlying genetical factors supporting the formation of multifocal systemic arterial aneurysms in addition to the known risk factors. The aim of this study was to identify disease-related genetic variants in patients with multiple arterial aneurysms.

## Materials and methods

Selection of the patients enrolled was described previously [5]. Patients with multiple aneurysms (at least four) diagnosed in a single center for vascular surgery from 2006 to 2016 were identified. From a total cohort of 3107 patients with arterial aneurysms identified by the ICD-10 (ICD-10-GM) classification system (code I71 and I72), 918 patients were excluded due to known hereditary disorders of connective tissue (e.g., Marfan syndrome, Ehlers-Danlos syndrome or Loeys-Dietz syndrome), vasculitis or other arterial pathologies than true aneurysms (e.g., dissection, intramural hematoma, penetrating aortic ulcer or false aneurysm). Of the remaining 2189 patients, 143 presented multiple aneurysms. The total number of aneurysms per patient was defined by the sum of affected dilated arterial segments throughout the entire arterial system. The institutional vascular biomaterial bank was screened for eligible samples, and nine peripheral blood samples of respective patients were available and further analyzed after DNA extraction [9].

Whole exome sequencing (WES) was performed on a Genome Analyzer IIx system (Illumina Inc., San Diego, CA, USA) after in-solution enrichment of exonic sequences (SureSelect Human All Exon 38 Mb kit, Agilent Technologies Germany GmbH & Co. KG, 76337 Waldbronn, Germany). Read alignment to the human genome assembly hg19 was performed with the Burrows-Wheeler Aligner software (version 0.5.8). Single-nucleotide variants (SNV) were detected with SAMtools (version 0.1.7), as described previously [10].

Possible candidate variants were selected based on in silico predictions: (I) Truncating variants (frameshift, splice acceptor, splice donor, start-lost, stop-lost and stop-gain) or (II) Variants that were classified as likely pathogenic (SIFT score < 0.05 or PolyPhen score > 0.9) and with low (< 0.001) or unknown gnomAD allele frequency were evaluated [11]. The human genome databases GeneCards and MalaCards were correlated with regard to a possible association with vascular diseases [12, 13].

The study protocol was approved by the institutional review board. Informed consent was obtained from all subjects involved in the study. The study adhered to the ‘Strengthening the Reporting of Observational studies in Epidemiology’ (STROBE) guidelines [14].

## Results

From the nine patients analyzed, a total of 24 variants in 23 different genes associated with vascular diseases were detected. Table 1 shows respective identified variants connected to vascular diseases accompanied by aneurysm count and location, age at sampling and age at initial aneurysm diagnosis of respective patients.

**Table 1:** Variants connected to vascular disorders

Patient	Aneurysm Count	Aneurysm Location	Age at Sampling (y)	Age at Initial Diagnosis (y)	Symbol	Chromo-some	Position	HGVSc	Conse-quence	Impact	Associated Vascular Disorder	MalaCard Relevance Score	Existing Variation	SIFT	PolyPhen	gnomADe_AF	gnomADgAF
#01	6	CIA(rl), IIA(rl), EIA(rl)	78	78	ELK3	chr12	96,247,660	ENST00000228741.8:c.928G > A	Missense	Moderate	AAA	1,1	rs762306653&COSV57386396	deleterious(0)	probably damaging(0.995)	2,02E-05	1,31E-05
					TULP3	chr12	2,939,337	ENST00000448120.7:c.1222 C > T	Missense	Moderate	AAA	1,0	rs140237163	deleterious(0)	probably damaging(1)	2,79E-05	5,91E-05
					TET2	chr4	105,235,547	ENST00000380013.9:c.1608dup	Frameshift	High	CV Disease	3,6					
					TET2	chr4	105,275,493	ENST00000380013.9:c.4983T > G	Stop	High	CV Disease	3,6	COSV54400022&COSV99485558				
					PPM1D	chr17	60,663,071	ENST00000305921.8:c.1337 C > A	Stop	High	CV Disease	0,9	CM131999&COSV59956020&COSV59958116				
#02	4	AbdAo, CIA(rl), CFA(r), PA(rl)	75	74	SH3TC1	chr4	8,240,935	ENST00000245105.8:c.3996del	Frameshift	High	TAAD	0,6	rs761148394			2,03E-05	3,29E-05
#03	4	AscAo, DFA(r), PA(rl)	70	70	SGCD	chr5	156,757,601	ENST00000337851.9:c.597dup	Frameshift	High	Aortic Dissection	1,0					
					GRK5	chr10	119,442,067	ENST00000392870.3:c.1036G > A	Missense	Moderate	Aortic Atherosclerosis	0,4		deleterious(0)	probably damaging(0.996)		
					GFRAL	chr6	55,351,579	ENST00000340465.2:c.697T > C	Missense	Moderate	CV Disease	0,2	rs746762126	deleterious(0)	probably damaging(1)	4,07E-06	6,57E-06
					TBC1D9	chr4	140,668,980	ENST00000442267.3:c.1525 C > T	Missense	Moderate	TAAD	0,5	rs759134525&COSV71307794	deleterious(0)	probably damaging(0.92)	1,61E-05	6,57E-06
#04	4	AscAo, CIA(r), IIA(r), PA(l)	81	76	FGGY	chr1	59,378,761	ENST00000303721.12:c.481del	Frameshift	High	Aortic Aneurysm	0,2					
					DGCR2	chr22	19,048,500	ENST00000263196.12:c.946G > C	Missense	Moderate	Ehlers-Danlos Syndrome	0,2	rs774733736	deleterious(0.01)	probably damaging(0.92)	1,99E-05	4,60E-05
#05	4	AbdAo, CIA(l), IIA(r), PA(r)	75	74	COL6A5	chr3	130,455,507	ENST00000373157.9:c.6631 C > T	Stop	High	Ehlers-Danlos-Syndrome	1,6	rs201040843			3,63E-05	1,98E-05
					JCAD	chr10	30,027,647	ENST00000375377.2:c.2501 A > C	Missense	Moderate	CAD and Atherosclerosis	4,9	rs554587601	deleterious(0)	probably damaging(0.984)	2,81E-05	2,63E-05
#06	8	AbdAo, CIA(rl), IIA(rl), SFA(rl), PA(l)	63	60	SCNN1D	chr1	1,287,185	ENST00000379116.10:c.1198del	Frameshift	High	TAAD	0,5	rs1432929546			4,03E-06	6,57E-06
					SLC27A6	chr5	128,966,197	ENST00000262462.9:c.61_65del	Frameshift	High	Aortic Valve Disease	0,2	rs540473562			0,002278	0,002366
					MYO9B	chr19	17,212,280	ENST00000682292.1:c.6450dup	Frameshift	High	Aortic Aneurysm	1,2	rs762474493			1,91E-05	1,97E-05
					AGBL1	chr15	86,269,927	ENST00000614907.3:c.1847 A > T	Missense	Moderate	CAD and Atherosclerosis	0,2		deleterious(0)	probably damaging(0.999)		
#07	5	AbdAo, CIA(rl), IIA(rl)	70	70	FCGBP	chr19	39,927,062	ENST00000616721.6:c.1300 C > T	Missense	Moderate	Aortic Aneurysm	0,2	rs369253964	deleterious(0)	probably damaging(0.968)	1,73E-05	3,29E-05
#08	8	ICA(rl), SubclA(rl), AbdAo, CIA(r), CFA(l), SFA(l)	62	40	SMAD3	chr15	67,181,355	ENST00000327367.9:c.773 A > C	Missense	Moderate	Loeys-Dietz syndrome 3	61,8		deleterious(0.01)	probably damaging(1)		
					PLD3	chr19	40,378,025	ENST00000409735.9:c.1325 C > T	Missense	Moderate	TAAD	0,6	rs762590435&COSV52520661	deleterious(0)	probably damaging(0.975)	1,60E-05	1,97E-05
#09	6	AbdAo, CIA(rl), CFA(r), PA(rl)	58	58	USP45	chr6	99,508,622	ENST00000500704.7:c.261G > T	Missense	Moderate	CAD and Atherosclerosis	0,2		deleterious(0)	probably damaging(1)		
					TNXB	chr6	32,096,845	ENST00000644971.2:c.1008 C > A	Missense	Moderate	Ehlers-Danlos Syndrome	58,8	rs754128472	deleterious(0)	probably damaging(0.994)	0	6,59E-06
					ACTN1	chr14	68,880,826	ENST00000394419.9:c.2117 C > T	Missense	Moderate	AAA	0,2	rs373795472&COSV51989436	deleterious(0.02)	probably damaging(0.999)	1,19E-05	1,97E-05

Variants with association to vascular diseases with aneurysm count and location, age at sampling in years and age at initial aneurysm diagnosis in years of respective patient. Relevance displayed by MalaCard relevance score (13). AscAo: Ascending Aorta; ICA: Internal Carotid Artery; SubclA: Subclavian Artery; AbdAo: Abdominal Aorta; CIA: Common Iliac Artery; IIA: Internal Iliac Artery; EIA: External Iliac Artery; CFA: Common Femoral Artery; DFA: Deep Femoral Artery; SFA: Superficial Femoral Artery; PA: Popliteal Artery; r: Right; l: Left; rl: Right and Left; HGVSc: HGVS Coding Sequence Name; AAA: Abdominal Aortic Aneurysm; TAAD: Thoracic Aortic Aneurysm and Dissection; CV: Cardiovascular; CAD: Coronary Artery Disease. SIFT: Sorting Intolerant From Tolerant; PolyPhen: Polymorphism Phenotyping; gnomADe_AF: Frequency of Existing Variant in gnomAD Exomes Combined Population; gnomADg_AF: Frequency of Existing Variant in gnomAD Genomes Combined Population

All patients were male. The aneurysm count ranged from 4 to 8 affected arterial locations with a median aneurysm count of 5 (interquartile range: 2). Affected aneurysm localizations were mainly the abdominal aorta as well as iliac, femoral, and popliteal arteries. Mean age at sampling was 70.2 (± 7.4) years, and mean age at initial aneurysm diagnosis was 66.7 (± 11.4) years. The detected variants comprised 14 missense variants, seven frameshift variants and three nonsense variants. For respective variants, associated vascular disorders were described, which included abdominal and thoracic aortic aneurysms and dissections, Ehlers-Danlos syndrome, coronary artery disease, aortic valve disease, and cardiovascular disease in general. Referring publications for each variant presented in Table 1 are listed in Supplemental Table 1. CNV analysis in all patients did not reveal any relevant findings. All identified nonsynonymous variants of all nine blood samples are presented in Supplemental Table 2.

One patient with eight aneurysms was heterozygous for a variant in *SMAD3*, for which pathogenic variants are phenotypically associated with Loeys-Dietz syndrome 3 (aneurysms-osteoarthritis syndrome). His age at initial aneurysm diagnosis was 40 years. In addition, a heterozygous variant in *TNXB* was found in one patient with five aneurysms. Biallelic pathogenic variants in *TNXB* are associated with Ehlers-Danlos syndrome (Classic-Like). The patient was 58 years old at initial aneurysm diagnosis. Another patient with six aneurysms was heterozygous for two variants in *TET2* (one frameshift and one nonsense variant) and one variant in *PPM1D*. Variants in those regulator and DNA repair genes are associated with clonal hematopoiesis of indeterminate potential (CHIP). The patient was 78 years old at initial aneurysm diagnosis.

## Discussion

Genetic testing and counselling is recommended for patients with thoracic aortic disease (TAD) younger than 60 years, positive family history, or coexistence of intracranial or peripheral aneurysms, as hereditary factors are suspected frequently in thoracic aortic pathologies [15]. We recently reported a high ratio of positive genetic variants in fibrillin (*FBN*) and actin alpha 2 gene (*ACTA2*) in preselected patients with Stanford type B aortic dissections (STBAD) [16]. This study here evaluated genetic variants in patients with a systemic appearance of multiple arterial aneurysms using whole exome sequencing. As STBAD and other multiple arterial dissections might affect mainly genes from the defined TAD gene panel, other genes might be involved in multiple arterial aneurysms (e.g., *TNXB*).

Systemic multiple aneurysms are either ascribed to pronounced atherosclerosis or heritable disorders, non-infectious autoimmune vasculitis and infectious disease affecting even younger age groups [17]. Known genetic causes for multiple arterial aneurysms include aneurysms-osteoarthritis syndrome, vascular Ehlers-Danlos syndrome, Marfan syndrome, Loeys-Dietz syndrome, arterial tortuosity syndrome or autosomal dominant polycystic kidney disease [18]. Genetic findings should be discussed with affected patients and family members should be tested to enable personalized follow up or screening investigations to reduce mortality and morbidity.

Analyzing nine patients with multiple aneurysms by WES we uncovered 23 putative disease-causing variants in genes known to be associated with arterial aneurysms, in particular in *SMAD3*,* TNBX*,* TET2* and *PPM1D*.

*SMAD3* encodes a member of the TGF-β pathway essential for signal transmission [19]. Pathogenic *SMAD3* variants lead to increased aortic expression of several key players in the TGF-β pathway and cause a syndromic form of aortic aneurysms and dissections with early-onset osteoarthritis [20]. Summarized under Loeys-Dietz syndrome 3 (previously known as aneurysms-osteoarthritis syndrome), and inherited in autosomal dominant fashion, affected patients present aortic aneurysms, arterial tortuosity, and other vascular disorders [21, 22]. In the ClinVar database, the *SMAD3* variant identified in this study is classified as of uncertain significance for familial thoracic aortic aneurysm and aortic dissection according to a single submission (American College of Medical Genetics [ACMG] guidelines) [23, 24]. *TNXB* encodes for Tenascin-X, an extracellular matrix glycoprotein for which homozygosity or compound heterozygosity of pathogenic variants result in classical-like Ehlers-Danlos syndrome [25]. Clinical features are joint hypermobility, hyperextensible velvety skin and easy bruising [26]. Moreover, vascular fragility such as rupture of AAA has been reported [25]. As tenascin-X deficiency is of recessive inheritance, the identified variant is not sufficient to determine pathogenicity [27]. Nevertheless, as described for vascular diseases in general, there might be the possibility of a somatic second hit during aneurysm formation, resulting in a somatic biallelic variant state [28], or a second germline variant is present but could not be uncovered due to limitation of WES. In the ClinVar database, the *TNXB* variant identified in this study is classified as of uncertain significance for cardiovascular phenotype according to a single submission (Ambry Variant Classification Scheme 2023, Ambry Genetics, Aliso Viejo, California, USA) [24].

Clonal hematopoiesis of indeterminate potential (CHIP) is defined as presence of an expanded somatic blood-cell clone in persons without other hematologic abnormalities [29]. It is common among older persons and associated with a 0.5-1.0% risk per year of leukemia and it confers a two-fold increase in cardiovascular risk independent of traditional risk factors [29, 30]. The most frequent mutated driver genes in CHIP are *DNMT3A*, *TET2*, and *ASXL1*, increasing the risk of atherosclerotic cardiovascular disease with a hazard ratio of 1.7, 1.9 and 2.0 respectively [30]. *TET2* plays an important role in restraining the expression of inflammatory genes in macrophages, whereas *PPM1D* is part of the DNA damage response pathway [30, 31]. Although CHIP was found to be a risk factor of atherosclerosis, stroke and myocardial infarction, and underlying pathomechanisms are known to be similar to those in aneurysm formation, a clinical association to arterial aneurysms is not yet described [32]. Nevertheless, Liu et al. identified *TET2* as master epigenetic regulator of smooth muscle cell (SMC) differentiation, which could potentially contribute to aneurysm formation [33]. Apart from the identified variants in *TET2* and *PPM1D*, no other variants in genes associated with CHIP according to the existing literature (*DNMT3A*, *ASXL1*, *JAK2*, *TP53*, *SF3B1*, or *SRSF2*) were detected in this study. In the ClinVar database, the identified variants in *TET2* and *PPM1D* were not classified. Besides, none of the other 19 identified variants were classified in the ClinVar database.

Management regarding aortic aneurysm repair in patients with hereditary connective tissue diseases require specific and individualized therapeutical strategies [15]. Patients with heritable thoracic aortic diseases (HTAD) caused by a highly penetrant (likely) pathogenic variant (e.g., Marfan, Loeys-Dietz or vascular Ehlers-Danlos syndrome) have a higher risk of aortic dissection or rupture. Such patients should be treated by an experienced multidisciplinary aortic team and seem to profit from surgical therapy at an earlier state of the disease compared to sporadic aortic diseases. For patients with Loeys-Dietz syndrome attributable to pathogenic variants in *TGFBR1*, *TGFBR2*, or *SMAD3*, surgical replacement of the aortic arch, descending aorta or abdominal aorta may be considered already at a diameter of ≥ 4.5 cm [15]. There is no specific guideline recommendation regarding the diameter threshold for prophylactic surgical intervention for aortic aneurysms in vascular Ehlers-Danlos syndrome [15, 34]. For patients with Marfan, Loeys-Dietz or vascular Ehlers-Danlos syndrome and an intact thoracoabdominal aortic aneurysm requiring intervention, open surgical repair is in general recommended over endovascular repair [15]. The underlying hypothesis for the potentially poorer outcome of endovascular procedures is thought to be an inappropriate landing zone due to vascular fragility, increasing the risk for endoleaks and reinterventions. If the landing zones for endovascular repair are represented by already implanted vascular grafts instead of native tissue, for example after open aortic arch and open abdominal aortic repair, endovascular treatment is preferred over additional open surgery [35]. In a recent retrospective multicenter study of endovascular aortic interventions in patients with connective tissue disease, Olsson et al. report high rates of early technical success (98.2%), low perioperative mortality (2.9%), and 5-year survival rates comparable with reports of open aortic surgery in respective patients (80.6% for patients with Marfan syndrome, 85.2% for patients with Loeys-Dietz syndrome, and 43.8% for patients with vascular Ehlers-Danlos syndrome), whereas the majority of patients were treated for aortic dissections (88.9%) and only 19 patients (11.1%) for degenerative aneurysms [36]. There are currently no specific recommendations on aneurysm repair strategies for patients with multiple arterial aneurysms.

Both patients with the *SMAD3* and *TNXB* variants in this study underwent endovascular aortic repair (EVAR) for their AAA, with simultaneous iliac side branches for aneurysm extension in the iliac arteries. In both cases, there was no diagnosis of a genetic disease at time of intervention and endovascular therapy was preferred to open surgery due to prior abdominal surgery in accordance with the respective guidelines [37]. The patient with the *SMAD3* variant recently showed complications in terms of endoleaks (Type 1a and 1b) with aneurysm sac expansion after 2.5 years of follow-up, requiring reintervention to prevent secondary aneurysm rupture. The patient with the *TNXB* variant does not present any complications within two years of follow-up. Of the seven other patients in this study, four received EVAR for their AAA, of which only the patient with the *TET2* and *PPM1D variants* developed a relevant endoleak (1b) with re-intervention five years after EVAR. The early endovascular failure and occurrence of significant endoleaks in the patient with the *SMAD3* mutation could be explained by genetically triggered aneurysm formation similar to known connective tissue diseases. As patients with hereditary connective tissue diseases present worse outcomes after endovascular aneurysm repair compared to open surgery, immediate genetical diagnosis in patients with multiple arterial aneurysms could be used for surgical decision making and to improve personalized medicine in vascular surgery [15].

All respective patients were informed and individualized follow up protocols were initiated according to recommendations for connective tissue disease with vascular complications [15]. These WES data provide information on rare genetical variants in patients with multiple arterial aneurysms. The other presented rare variants need further validation in other cohorts with vascular pathologies.

As the respective patients were recruited from the clinical setting of the department of vascular and endovascular surgery, no detailed assessment of dysmorphisms or other clinical genetic assessment was performed. The data presented in this study suggest greater than expected contribution of germline genetic predisposition to arterial aneurysm disease, and favor the idea of joint clinical assessments and interdisciplinary clinics of vascular surgery and clinical genetics.

Patients with multiple arterial aneurysms should be considered for genetic testing. In addition, further analysis of CHIP mutations should be considered for patients with arterial aneurysms. Two variants of uncertain significance (VUS) were detected in this study. Further investigation into the causes of multiple arterial aneurysms is of great importance and could help to identify the predispositions and pathomechanisms of this complex group of diseases.

## Conclusions

Patients with multiple arterial aneurysms and young age at disease onset should be considered for genetic diagnostic testing. If gene panel testing doesn’t identify pathogenic variants, further comprehensive genetic testing using WES may be suggested. We recommend consideration of genetic diagnostic testing and counselling in patients with multiple aneurysms and dissections to rule out syndromic hereditary connective tissue diseases as these findings demand patient-specific disease management. The current data basis and knowledge of the specific variants is still insufficient for variant classification at the present time. This underlines the need for a better understanding of the consequences of genetic variants, especially in the clinical context of multifocal vascular diseases.

## Electronic supplementary material

Below is the link to the electronic supplementary material.

Supplementary Material 1

Supplementary Material 2

## Data Availability

The data that support the findings of this study are available on request from the corresponding author.
