# Supplementary material for: Genetic variants in patients with multiple arterial aneurysms
Source: Langenbecks Arch Surg. 2024 Oct 9;409(1):304. doi: 10.1007/s00423-024-03488-5 (PMC11464538; doi:10.1007/s00423-024-03488-5)
Supplement: Supplementary file 1 — Supplementary Material 1 [file 423_2024_3488_MOESM1_ESM.docx]

**Supplemental Table 1:** Referring publications of presented variants

| **Gene-Symbol** | **Associated Publication** |
| --- | --- |
| *ELK3* | Yang K, Cui S, Wang J, et al. Early Progression of Abdominal Aortic Aneurysm is Decelerated by Improved Endothelial Barrier Function via ALDH2-LIN28B-ELK3 Signaling. *Adv Sci (Weinh)*. 2023;10(32):e2302231. doi:10.1002/advs.202302231 |
| *TULP3* | Cai B, Yang B, Huang D, et al. STAT3-induced up-regulation of lncRNA NEAT1 as a ceRNA facilitates abdominal aortic aneurysm formation by elevating TULP3. *Biosci Rep*. 2020;40(1):BSR20193299. doi:10.1042/BSR20193299 |
| *TET2* | Marnell CS, Bick A, Natarajan P. Clonal hematopoiesis of indeterminate potential (CHIP): Linking somatic mutations, hematopoiesis, chronic inflammation and cardiovascular disease. J Mol Cell Cardiol. 2021;161:98-105. doi:10.1016/j.yjmcc.2021.07.004 |
| *PPM1D* | Marnell CS, Bick A, Natarajan P. Clonal hematopoiesis of indeterminate potential (CHIP): Linking somatic mutations, hematopoiesis, chronic inflammation and cardiovascular disease. J Mol Cell Cardiol. 2021;161:98-105. doi:10.1016/j.yjmcc.2021.07.004 |
| *SH3TC1* | Prakash SK, LeMaire SA, Guo DC, et al. Rare copy number variants disrupt genes regulating vascular smooth muscle cell adhesion and contractility in sporadic thoracic aortic aneurysms and dissections [published correction appears in Am J Hum Genet. 2013 Jun 6;92(6):1013]. *Am J Hum Genet*. 2010;87(6):743-756. doi:10.1016/j.ajhg.2010.09.015 |
| *SGCD* | Ainiwan M, Wang Q, Yesitayi G, Ma X. Identification of FERMT1 and SGCD as key marker in acute aortic dissection from the perspective of predictive, preventive, and personalized medicine. *EPMA J*. 2022;13(4):597-614. Published 2022 Nov 14. doi:10.1007/s13167-022-00302-4 |
| *GRK5* | Wu JH, Zhang L, Fanaroff AC, et al. G protein-coupled receptor kinase-5 attenuates atherosclerosis by regulating receptor tyrosine kinases and 7-transmembrane receptors. *Arterioscler Thromb Vasc Biol*. 2012;32(2):308-316. doi:10.1161/ATVBAHA.111.239608 |
| *GFRAL* | Xiao QA, He Q, Zeng J, Xia X. GDF-15, a future therapeutic target of glucolipid metabolic disorders and cardiovascular disease. *Biomed Pharmacother*. 2022;146:112582. doi:10.1016/j.biopha.2021.112582 |
| *TBC1D9* | Prakash SK, LeMaire SA, Guo DC, et al. Rare copy number variants disrupt genes regulating vascular smooth muscle cell adhesion and contractility in sporadic thoracic aortic aneurysms and dissections [published correction appears in Am J Hum Genet. 2013 Jun 6;92(6):1013]. *Am J Hum Genet*. 2010;87(6):743-756. doi:10.1016/j.ajhg.2010.09.015 |
| *FGGY* | Chai T, Tian M, Yang X, Qiu Z, Lin X, Chen L. Association of Circulating Cathepsin B Levels With Blood Pressure and Aortic Dilation. *Front Cardiovasc Med*. 2022;9:762468. Published 2022 Mar 29. doi:10.3389/fcvm.2022.762468 |
| *DGCR2* | Niederhoffer KY, Fahiminiya S, Eydoux P, et al. Diagnosis of Van den Ende-Gupta syndrome: Approach to the Marden-Walker-like spectrum of disorders. *Am J Med Genet A*. 2016;170(9):2310-2321. doi:10.1002/ajmg.a.37831 |
| *COL6A5* | Martinelli-Boneschi F, Colombi M, Castori M, et al. COL6A5 variants in familial neuropathic chronic itch. *Brain*. 2017;140(3):555-567. doi:10.1093/brain/aww343 |
| *JCAD* | Douglas G, Mehta V, Al Haj Zen A, et al. A key role for the novel coronary artery disease gene JCAD in atherosclerosis via shear stress mechanotransduction. *Cardiovasc Res*. 2020;116(11):1863-1874. doi:10.1093/cvr/cvz263 |
| *SCNN1D* | Prakash SK, LeMaire SA, Guo DC, et al. Rare copy number variants disrupt genes regulating vascular smooth muscle cell adhesion and contractility in sporadic thoracic aortic aneurysms and dissections [published correction appears in Am J Hum Genet. 2013 Jun 6;92(6):1013]. *Am J Hum Genet*. 2010;87(6):743-756. doi:10.1016/j.ajhg.2010.09.015 |
| *SLC27A6* | Chen MC, Chang JP, Lin YS, et al. Deciphering the gene expression profile of peroxisome proliferator-activated receptor signaling pathway in the left atria of patients with mitral regurgitation. *J Transl Med*. 2016;14(1):157. Published 2016 Jun 2. doi:10.1186/s12967-016-0871-3 |
| *MYO9B* | Ma D, Zheng B, Suzuki T, et al. Inhibition of KLF5-Myo9b-RhoA Pathway-Mediated Podosome Formation in Macrophages Ameliorates Abdominal Aortic Aneurysm. *Circ Res*. 2017;120(5):799-815. doi:10.1161/CIRCRESAHA.116.310367 |
| *AGBL1* | Tang W, Schwienbacher C, Lopez LM, et al. Genetic associations for activated partial thromboplastin time and prothrombin time, their gene expression profiles, and risk of coronary artery disease. *Am J Hum Genet*. 2012;91(1):152-162. doi:10.1016/j.ajhg.2012.05.009 |
| *FCGBP* | Gäbel G, Northoff BH, Weinzierl I, et al. Molecular Fingerprint for Terminal Abdominal Aortic Aneurysm Disease. *J Am Heart Assoc*. 2017;6(12):e006798. Published 2017 Nov 30. doi:10.1161/JAHA.117.006798 |
| *SMAD3* | van de Laar IM, Oldenburg RA, Pals G, et al. Mutations in SMAD3 cause a syndromic form of aortic aneurysms and dissections with early-onset osteoarthritis. Nat Genet. 2011;43(2):121-126. doi:10.1038/ng.744 |
| *PLD3* | Prakash SK, LeMaire SA, Guo DC, et al. Rare copy number variants disrupt genes regulating vascular smooth muscle cell adhesion and contractility in sporadic thoracic aortic aneurysms and dissections [published correction appears in Am J Hum Genet. 2013 Jun 6;92(6):1013]. *Am J Hum Genet*. 2010;87(6):743-756. doi:10.1016/j.ajhg.2010.09.015 |
| *USP45* | Yamada Y, Yasukochi Y, Kato K, et al. Identification of 26 novel loci that confer susceptibility to early-onset coronary artery disease in a Japanese population. *Biomed Rep*. 2018;9(5):383-404. doi:10.3892/br.2018.1152 |
| *TNXB* | Okuda-Ashitaka E, Matsumoto KI. Tenascin-X as a causal gene for classical-like Ehlers-Danlos syndrome. Front Genet. 2023;14:1107787. Published 2023 Mar 15. doi:10.3389/fgene.2023.1107787 |
| *ACTN1* | Cao GM, Xuan XZ, Dong HL. Low expression of integrin signaling pathway genes is associated with abdominal aortic aneurysm: a bioinformatic analysis by WGCNA. *Eur Rev Med Pharmacol Sci*. 2022;26(8):2847-2860. doi:10.26355/eurrev_202204_28615 |
